# Advancing individual finger classification through a sandwich enhanced CBAM network with ultra-high-density EEG data

**DOI:** 10.3389/fnhum.2026.1751058

**Published:** 2026-02-18

**Authors:** Xinguo Zhang, Yiman Zhang, Hong Peng, Tao Deng

**Affiliations:** 1 Key Laboratory of China's Ethnic Languages and Information Technology of Ministry of Education, Chinese National Information Technology Research Institute, Northwest Minzu University, Lanzhou, China; 2 School of Artificial Intelligence, Hebei Institute of Communications, Shijiazhuang, China; 3 School of Information Science and Engineering, Lanzhou University, Lanzhou, China; 4 School of Mathematics and Computer Science, Northwest Minzu University, Lanzhou, China

**Keywords:** attention mechanism, brain-computer interfaces, Convolutional Block Attention Module, individual finger classification, ultra-high-density EEG

## Abstract

**Introduction:**

Ultra-High-Density Electroencephalography (uHD EEG) has gained increasing attention for its potential in individual finger decoding. However, accurately classifying these movements remains challenging due to the subtle spatial overlaps in cortical activity, which standard architectures often fail to isolate.

**Methods:**

To address this, we propose the Sandwich enhanced Convolutional Block Attention Module (SCBAM). The unique sandwich structure integrates dual attention mechanisms between convolutional layers, enabling the network to more effectively refine high-dimensional spatial features.

**Results and discussion:**

The proposed network achieves an average accuracy of 78.63 (1.56)% in binary classification across ten finger pairs in five subjects, with the highest accuracy of 85% obtained at Thumb vs. Ring. The proposed network achieves an average accuracy of 61.12 (0.95)% in five-class classification across five subjects, with a highest accuracy of 62.36% on subject S2. The five-class classification is performed using 10 binary classifiers under a one-vs.-one strategy. Notably, five-class classification of individual fingers has not been extensively explored in the current literature, particularly with high-density EEG (HDEEG) data. This study addresses this gap, offering a valuable reference for future discussions. We conduct ablation studies to investigate the individual and synergistic effects of the modules in the proposed model. The results highlight the effects of two sequential attention mechanisms in this task. We conduct comparative experiments of our proposed model against six benchmark networks. The results from SCBAM significantly outperform these established models with FBCSP features. The proposed SCBAM significantly improves accuracy in binary finger classification compared to SVM and MLP using the same uHD EEG dataset. In summary, this study presents a high-performance hybrid network for individual finger classification and highlights the potential of uHD EEG for dexterous task decoding in Brain-Computer Interfaces (BCI).

## 1 Introduction

Brain-computer interface (BCI) offers a promising communication solution for individuals with severe neuromuscular disorders (Wolpaw et al., 2002). A main task of BCI is motor imagery (Pfurtscheller et al., 2000). These systems provide high temporal resolution but offer limited spatial resolution that constrains the precise localization of neural sources (Pfurtscheller et al., 1997). Traditional EEG systems are according to standard layouts (e.g., 10-20 or 10-10 systems), with inter-electrode distances ranging roughly from 20 mm to 60 mm (Chatrian et al., 1985; Jasper, 1958), brain rhythms (alpha, beta, and gamma). Event-related synchronization and desynchronization are widely applied in motor imagery decoding (Pfurtscheller and Lopes da Silva, 1999). EEG decoding has been recognized as a pattern recognition task for decades (Krusienski et al., 2006). Spatial filters, such as ear-reference, common average reference (CAR), small Laplacian, and large Laplacian, have been widely applied in this context (McFarland et al., 1997).

The pioneering study of Müller-Gerking et al. (1999) introduced CSP as a feature extraction method for single-trial EEG classification in movement tasks. Lotte and Guan (2011) unified different kinds of regularization CSP methods, which addressed problems of overfitting and improving generalization. Combining with the frequency band of the EEG signal, the Filter Bank Common Spatial Pattern (FBCSP) algorithm was shown to have superior classification accuracies on various datasets, including BCI Competition IV Datasets 2a and 2b (Ang et al., 2008; Quadrianto et al., 2007). The classification algorithms in the field of Brain-Computer Interface (BCI) have evolved significantly over the last two decades. According to a 2007 review (Lotte et al., 2007), the popular classifiers of that time included linear models, such as Linear Discriminant Analysis (LDA) and Support Vector Machines (SVM). Currently, the trend of classifier algorithms has shifted toward deep neural networks due to advancements in computational power and the availability of large datasets (Lotte et al., 2018).

The EEG signal has achieved significant accuracy in decoding prominent movements, such as the left hand, right hand, foot, and tongue, over the past few decades (McFarland et al., 2000; Lange et al., 2016). However, decoding dexterous individual finger movements has shown limited accuracy (Liao et al., 2014; Paek et al., 2014). For individual finger movements, where ECoG signals were considered more feasible (Liao et al., 2014). ECoG signals demonstrate high spatial resolution and a high signal-to-noise ratio. Researchers have put efforts into unveiling the properties of ECoG during motor tasks for decades (Volkova et al., 2019; Wu et al., 2024). High frequency components such as gamma and super gamma rhythms, together with the low frequency rhythms such as delta, alpha, and beta, have also been widely applied in decoding (Kubánek et al., 2009). The properties of ECoG rhythms–specifically phase (Miller et al., 2012), power (Miller K. J. et al., 2009), and modulation (Miller K. et al., 2009)—have been extensively explored in motor decoding tasks. The individual fingers' continuous flexion and extension trajectory decoding from human ECoG is a topic in BCI competition IV (Tangermann et al., 2012). The discrete classification of individual finger flexion decoding was reported in Shenoy et al. (2007), Kubánek et al. (2009), and Chestek et al. (2013). However, ECoG collection requires craniotomy to place electrodes on the surface of the cortex, making the procedure high-risk and limiting its use in daily applications (Miller et al., 2007; Hotson et al., 2016; Jiang et al., 2018).

Ultra-high-density EEG (uHD EEG) enhances spatial resolution by using a significantly larger number of electrodes (>256) across the scalp (Krishnan et al., 2018; Ma et al., 2022). This increased density allows for more precise localization of brain activity and detection of subtle neural signals, with applications in both clinical (Kumar et al., 2018) and research settings (Schreiner et al., 2023). These advances facilitate decoding of individual finger movements (Liao et al., 2014), which are challenging or impossible with conventional EEG (Schreiner et al., 2024). Lee et al. (2022) and Nemes et al. (2024) reported the binary classification accuracy of ten individual finger pairs. The study in Lee et al. (2022) achieved an average accuracy of 64.8% using Support Vector Machines (SVM), while Nemes et al. (2024) attained an average accuracy of 65.68% with Multi-Layer Perceptron (MLP). These results reveal there is still room for improvement in binary finger classification. Furthermore, to our knowledge, there is currently no literature addressing the five-class classification of the individual fingers with uHD EEG data, indicating a research gap in this area.

Deep learning has advanced the analysis and classification in BCI. Deep learning networks, such as Convolutional Neural Networks (CNN) (LeCun and Bengio, 1998), Recurrent Neural Networks (RNNs) (Sherstinsky, 2018), and attention mechanisms (Vaswani et al., 2017; Park et al., 2018), have been successfully used for neural decoding (Yao et al., 2024). Deep convolutional networks (ConvNets) are capable of extracting multiple levels of feature directly from raw EEG signals (Kern et al., 2019). Bashivan et al. (2016) developed the EEGLearn network, drawing inspiration from the popular Visual Geometry Group (VGG) architecture in the image processing field, which was then applied to the context of the mental load task. Lawhern et al. (2018) proposed EEGNet, which utilizes the temporal and spatial information from EEG signals through two convolutional layers and implements depthwise convolution in the spatial layer by assigning channel-wise filters. Schirrmeister et al. (2017) designed a shallow and a deep ConvNets for classifying movements of the left hand, right hand, foot, and rest with BCI Competition IV 2a and 2b EEG data. Shallow ConvNets combined with intermediate FBCSP features offer advantages of lightweight architecture, fewer parameters, and reduced tuning time, ultimately achieving comparable results (Schirrmeister et al., 2017). The evolution of attention mechanisms has significantly advanced the capabilities of neural network architectures. Initially, attention mechanisms (Vaswani et al., 2017) allowed models to selectively focus on specific parts of input data, enhancing their ability to capture relevant features in natural language processing (NLP). This was further developed by the Convolutional Block Attention Module (CBAM) (Woo et al., 2018), a lightweight module that operates on intermediate feature maps for image processing. The key characteristic of CBAM is its ability to process channel and spatial information sequentially through two separate attention modules.

The combination of convolutional layers and attention mechanisms has been applied for EEG decoding tasks. Eldele et al. (2021) present a novel approach that combines a multi-resolution convolutional neural network (MRCNN) with a multi-head transformer for sleep stage classification from single-channel EEG data. They named their model the attensleep network. Shi et al. (2023) come up with a Multi-band EEG Transformer (MEET) model for brain state classification. In the MEET, a multi-head attention mechanism is applied across multiple frequency bands of images of EEG signals. The fusion of these frequency band images enhances MEET's ability to discern brain states. In a recent study reported by Ma et al. (2024), a model combining a two-branch 3D convolutional network and a temporal attention mechanism has been proposed and applied to the motor imagery task with the BCI Competition IV 2a and 2b dataset. Song et al. (2023) proposed the EEG Conformer, a unified architecture that leverages CNNs for local feature learning and Transformers for global correlation extraction to improve EEG signal decoding. Liu et al. (2024) introduce MSVTNet, a novel end-to-end multi-scale vision transformer that integrates CNN-based local spatio-temporal extraction with multiscale feature tokenization to enhance MI-EEG classification. Deep Neural Networks (DNNs) are increasingly applied and crucial for advancing Brain-Computer Interface (BCI) technologies and rehabilitation devices (Wu et al., 2024). The main contributions of this study are as follows:

- This study presents a network to decode individual finger movements from uHD EEG data. We achieve an average accuracy of 78.63% in binary classification among 10 finger pairs and 61.12% in five-class classification across 5 subjects. To our knowledge, this is the first attempt at five-class classification of individual finger movements using uHD EEG, demonstrating the feasibility of this approach for the task.
- We propose a model integrating convolutional layers and Convolutional Block Attention Module (CBAM) for this task. The model begins with a convolutional layer for low-level features, followed by two attention mechanisms that process preceding filters, spatial, and rhythm domain information. This model is completed by an additional convolutional layer for high-level feature extraction. We name this network Sandwich enhanced CBAM (SCBAM). Additionally, we conduct ablation studies to evaluate the contribution of each module and to analyze the synergistic effects of multiple modules.
- We process the uHD EEG data with the Common Average Reference (CAR) method and utilize the FBCSP for feature extraction. The extracted features after the FBCSP are then fed into our proposed model for classification.
- Comparative experiments are conducted between our SCBAM model and several state-of-the-art networks, including EEGNet (Lawhern et al., 2018), EEGLearn (Bashivan et al., 2016), Shallow ConvNets (Schirrmeister et al., 2017), Deep ConvNets (Schirrmeister et al., 2017), EEG Conformer (Song et al., 2023), and Msvt Net (Liu et al., 2024) to evaluate the effectiveness of the proposed model.
- We compare our classification results obtained from uHD EEG data with results gained from current literature of the same task, including those decoded from uHD EEG, EEG, and ECoG. This analysis demonstrates that uHD EEG is feasible and has the potential to improve performance on dexterous movement tasks.

The rest of this study is organized as follows: Section 2 illustrates the methodology of data processing and network design. Section 3 presents experimental results and analysis, and Section 4 discusses the proposed networks' performance and outlines future work. Finally, Section 5 provides a comprehensive conclusion to the study.

## 2 Materials and methods

**Figure 1:**
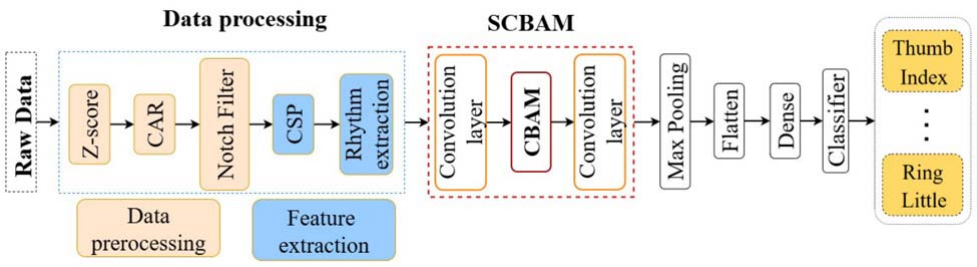
The workflow of the uHD EEG.

In this section, we reiterate the collecting experiments of the uHD EEG dataset from the (Lee et al., 2022). Then, we detail the classification method employed for uHD EEG. To enhance signal quality, we utilize a Common Average Reference (CAR) for spatial filtering and apply a notch filter to eliminate power line noise. We apply the Filter Bank Common Spatial Pattern (FBCSP) for feature extraction. We design an innovative network to perform classification tasks in two scenarios. In the binary classification scenario, we classify 10 movement pairs generated by 5 fingers. For the five-class classification scenario, we build upon the 10 binary classifiers using a one-vs-one strategy with majority voting. We conduct ablation experiments to investigate the contributions of the components in the proposed network. We conduct rigorous performance evaluations on various architectures and state-of-the-art models, including EEGNet (Lawhern et al., 2018), EEGLearn (Bashivan et al., 2016), Shallow ConvNets (Schirrmeister et al., 2017), Deep ConvNets (Schirrmeister et al., 2017), EEG Conformer (Song et al., 2023), and Msvt Net (Liu et al., 2024). The workflow of the uHD EEG is illustrated in Figure 1.

### 2.1 uHD EEG dataset

The uHD EEG data collection was approved by the Institutional Review Board of Korea Advanced Institute of Science and Technology (KH2018-127), and the gained dataset was published on OSF (Lee et al., 2022). The uHD EEG was collected from a finger movement experiment involving five healthy subjects (S1-S5). The participants used their dominant hands for testing, whereas S1 was left-handed and S2-S5 were right-handed. Each participant completed 10 sequential runs. Each run began with a 30-second baseline period, followed by alternating rest and finger extension periods. The rest period lasted between 3 and 4 s, while the extension (task) period lasted 5 s. During the task, participants extended the finger indicated by a colored cue displayed on a screen. Each finger was cued five times in a pseudo-random order, resulting in 25 finger extensions per run. Researchers use a novel g. Pangolin system to measure brain signal. The system achieves high spatial resolution, with 16 electrode grids (16 electrodes for each grid, 256 electrodes in total) placed over the contralateral sensorimotor cortex. The electrode grids were attached sequentially in the downward direction laterally of the head, starting with the first electrode grid at the Cz position. Dense distribution and small-sized electrodes result in an inter-electrode distance of 8.6 mm (uHD EEG). The sampling rate is 600 Hz, leading to 3,000 samples of each extension trail. The high number of electrodes and the high sample rate ensure that the data contain rich spatial and temporal information. This high sample rate also captures sufficient frequency bands for brain rhythms, which are key information carriers. Thus, the collected uHD EEG data encompass multi-domain information.

### 2.2 Data processing

The data processing consists of two stages: preprocessing and feature extraction. In preprocessing, the raw data are initially standardized using a z-score, and then undergo common average referencing (CAR) to reduce interference from neighboring electrodes. Subsequently, a 4th-order Butterworth notch filter cascade is applied to eliminate interference of line power at 60 Hz and its harmonics (120 Hz, 180 Hz, and 240 Hz). We apply the FBCSP to extract spatial and frequency features. The FBCSP includes two steps:

Firstly, we utilize the Common Spatial Pattern (CSP) method to decompose the joint covariance matrices of the uHD EEG signals from two classes. CSP identifies spatial filters that lead to optimal discrimination between two classes:


$$\begin{array}{l} J\left(w\right)=\frac{w^{T}C_{1}w}{w^{T}C_{2}w} \end{array}$$ (1)

**Figure 2:**
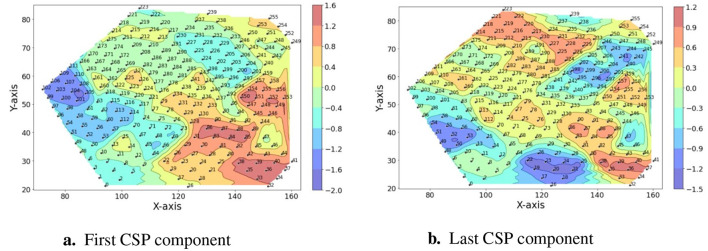
The topology patterns of the CSP components. **(a)** The pattern of the first CSP component. **(b)** The pattern of the last CSP component. The X-axis and Y-axis represent the medial-lateral and anterior-posterior directions of the human scalp, with the coordinates indicating the locations of each electrode. The color bar illustrates the value associated with each electrode, where red represents positive values and blue represents negative values. The intensity of the color indicates the absolute values, with high intensity representing large absolute values.

Specifically, the CSP components refer to the eigenvector corresponding to the large eigenvalue of the joint covariance matrix ($\frac{C_{1}}{C_{2}}$) associated with the first class, while the last CSP component corresponds to the small eigenvalue of the joint covariance matrix reflecting the large eigenvalue of the second class. To determine the optimal number of CSP components, experiments are conducted with the number of components ranging from 2 to 20. Ultimately, it is determined that a 12 (6 pairs) CSP components provides the best performance for the classification tasks, with 6 components corresponding to the first class and 6 to the second class for binary classification. Two CSP component patterns for movement pairs (thumb vs ring) are depicted in Figure 2 as an example.

Secondly, multiple 4th-order Butterworth band-pass filters are utilized to extract EEG rhythms: delta (1–4 Hz), alpha (8–12 Hz), beta (12–30 Hz), gamma (30–50 Hz), high gamma (75–115 Hz), and super high gamma (125–150 Hz). For CSP filtered rhythms, the centered mean power is calculated by subtracting the respective mean, squaring to get the power. The powers are log-transformed to enhance Gaussianity. Automated artifact rejection was performed to ensure signal integrity. For each channel, data segments with Z-scores greater than 6 were marked as artifacts. Entire experimental runs were rejected if more than 10% of the channels were classified as bad. All preprocessing parameters (*z*-score μ/σ), and all label-supervised spatial filters FBCSP were fitted using training runs only (runs 1-8) and then applied unchanged to test runs (runs 9–10). Hyperparameters were selected using validation within the training runs; test runs were used only once for final reporting. Notably, the percentage of data reduction from FBCSP is approximately 28%, and the output of the FBCSP will be fed into the proposed network.

### 2.3 Network design

**Figure 3:**
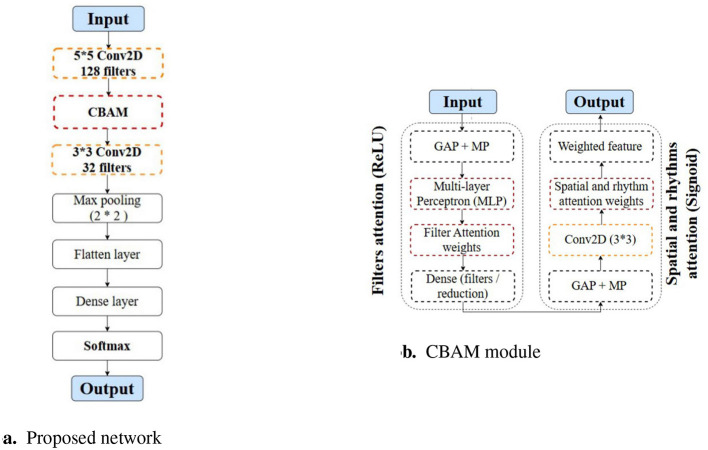
The flowchart of the proposed network and CBAM module. **(a)** The flowchart of the proposed network. **(b)** The workflow of the CBAM. CBAM, Convolutional Block Attention Module; GAP, Global Average Pooling; MP, Max Pooling.

**Figure 4:**
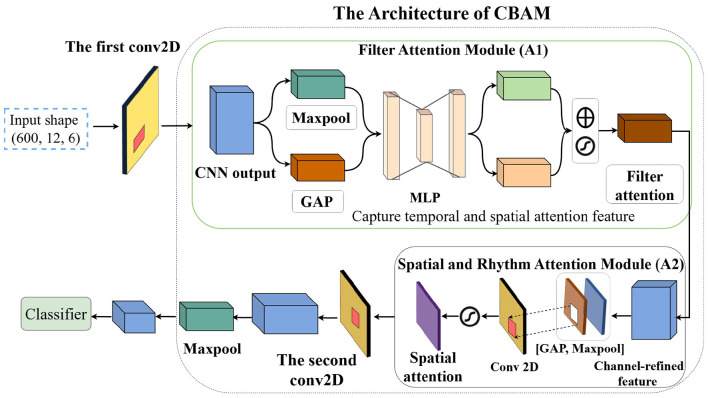
The architecture of the proposed network.

In this study, we investigate the network performance by integrating convolutional layers with multiple current popular attention mechanisms, including self-attention mechanism, the Convolutional Block Attention Module (CBAM), and multi-head transformers. We define the network in a sandwich configuration, placing convolutional layers both before and after the attention modules to facilitate multi-scale feature extraction and summarization. For the CBAM module, which contains two sequential attention mechanisms, with the first attention mechanism deals with the filter domain information from the preceding convolutional layer, and the second attention mechanism processes 2D information from the spatial and rhythm domains. We then switch the two attention modules in CBAM to study the effect of the order by placing the first one to handle 2D spatial and rhythm information, and the second to process filter information. We also conduct a thorough investigation into the optimal number of convolutional layers surrounding the CBAM module by adding layers both before and after the CBAM. The classification results indicate that increasing the number of convolutional layers does not yield any significant benefits for final accuracy. We name the winning model as Sandwich enhanced CBAM (SCBAM). The flowchart of the SCBAM is indicated in Figure 3. The CBAM module is treated as a component in Figure 3a of the overall data flow, and the details of the CBAM are then elaborated in Figure 3b. Figure 4 presents the structure and computational process within the network. It should be noted that the input of the SCBAM network is the features extracted from FBCSP.

#### 2.3.1 Network architecture and parameters

This SCBAM effectively integrates convolutional layers with the attention mechanisms by structuring the model to begin and end with convolutional layers surrounding the CBAM. The first important decision is how to represent the input *X*^*j*^, where *j* is the number of trials. To this end, we represent the input as *X*^*j*^ ∈ 𝕀^*T*·*C*·*R*^ 3D-array with the number of time samples is *T*, the number of CSP components *C*, and the number of rhythms *R*. The first convolutional layer performs 2D convolution across the time domain and spatial dimensions (CSP components) using 128 filters with a kernel size of (5, 5) and a stride of (1, 1). Filters are applied to slide the data to capture temporal and spatial information. The output feature map will be fed into the CBAM to further extract fine-scale features.

An intermediate feature map (128, 12, 6) as input for the subsequent CBAM module. The first attention mechanism in CBAM generates a 1D attention map for the filter domain. The second attention mechanism generates a 2D attention map hyperplane expanded by the spatial (CSP components) and rhythm domains. The computation of the two attention modules is as follows:


$$\begin{array}{l} \begin{array}{c} M^{\prime }=A_{f}\left(M\right)⊗M,\\ M^{\prime \prime }=A_{cr}\left(M^{\prime }\right)⊗M^{\prime }. \end{array} \end{array}$$ (2)

where *M* represents the input feature map, *A*_*f*_ is the attention map derived from the filter domain, generated by the preceding convolutional layer, *A*_*cr*_ is the attention feature map that is expanded by the CSP components and rhythm domain. The refined outputs are *M*′ and *M*″, ⊗ denotes the element-wise multiplication. Notably, the *A*_*cr*_ is a 2D attention map where rhythm domain information is first extracted.

The filter attention mechanism generates a 1D attention map through a global average pooling (GAP) and a max pooling (MP) layers, followed by a multi-layer perceptron (MLP). The GAP and MP operations generate average-pooled features and max-pooled features: $M_{\text{gap}}^{f}$ and $M_{\text{max}}^{f}$ respectively. Both descriptors are then fed into a MLP module to produce an attention map $M_{f}\in {\mathbb{R}}^{F\times 1\times 1}$. The MLP is composed of a hidden layer and a dense layer. To reduce parameter overhead, the hidden layer reduces the dimensionality by a reduction ratio *r* = 16, leading to a hidden size ℝ^*F*/*r*×1×1^. Specifically, the 128 input filters are first compressed to 8 and then reconstructed to 128 to generate filter-wise weights via a sigmoid function. The dense layer restores the original size 128 and applies a sigmoid activation function to generate attention weights. The filter attention is computed as:


$$\begin{array}{l} \begin{array}{c} M_{f}=\sigma \left(MLP\left(GAP\left(M\right)\right)+MLP\left(MP\left(M\right)\right)\right)\\ =\sigma \left(W_{1}\left(W_{0}M_{\text{gap}}^{f}\right)+W_{1}\left(W_{0}M_{\text{max}}^{f}\right)\right). \end{array} \end{array}$$ (3)

where the σ is the sigmoid activation function, *W*_0_ and *W*_1_ are the weights of MLP layers.

In the second attention mechanism, we first apply GAP and MP oprations, then concatenate the results to create an efficient feature descriptor $M_{\text{gap}}^{s}\in {\mathbb{R}}^{1\times C\times R}$. This attention module generates a 2D attention map via a Conv 2D layer, using 32 filters with a kernel size of (3, 3) and a stride of (1, 1). The second attention is computed as :


$$\begin{array}{l} \begin{array}{c} M_{cr}=\sigma \left(f^{3\times 3}\left(\left[GAP\left(M\right);MP\left(M\right)\right]\right)\right)\\ =\sigma \left(f^{3\times 3}\left(\left[M_{\text{gap}}^{s};M_{\text{max}}^{s}\right]\right)\right), \end{array} \end{array}$$ (4)

where the σ is the sigmoid activation function, *f*^3×3^ denotes the convolution operation.

After the attention modules, another convolutional layer is introduced for final feature map refinement. This layer utilizes 32 filters with a kernel size of (3, 3), a stride of (1, 1), and employs a ReLU activation function. This layer processes the features enhanced by the attention mechanisms from the filter, spatial, and rhythm domains. After that, a max-pooling layer with a pool size of (2, 2) and a stride of (2, 2) is applied to reduce computational complexity. With subsequent flattening and dense layers, this network effectively provides the discriminative feature maps for the final classification task. Besides, we use a dropout layer with a dropout rate of 0.3 to mitigate overfitting.

#### 2.3.2 Ablation studies

We conduct ablation experiments on the SCBAM model to assess the isolated effect of each key component by systematically removing: the first convolutional layer, the two attention modules in CBAM, respectively, and the second convolutional layer. To assess the effects of the first convolutional layer, we evaluate the results of the CBAM module alongside the second convolutional layer (CBAMs). For the second convolutional layer, we examine the results by combining the first convolutional layer with the CBAM module (fCBAM). For attention mechanisms, we keep the sandwich shape generated by the two convolutional layers and combine it with the filter attention module (SA1) and spatial and rhythm attention module (SA2), respectively. To explore the cooperation of multiple components, we investigate the performance of the CBAM module to study the synergistic effects of the two attention mechanisms, as well as a network comprising only two convolutional layers (two convs).

To systematically evaluate the contribution of individual data processing components and ensure a rigorous performance analysis, we conducted two targeted ablation studies. Specifically, we assessed the impact of Filter Bank Common Spatial Pattern (FBCSP) and Common Average Reference (CAR) by removing them individually from the pipeline.

### 2.4 Training and classification

In this section, we provide details of the training process and the classification strategy. Given that the number of trials is relatively small for effective tuning of a deep neural network, we chop the trials from the 3000 samples into 600 samples per trial, with a 50% (300) overlap. We concatenate the chopped trials from 10 runs, resulting in a total of 2,250 trials, 80% of which are used for training and 20% for testing. To guarantee the separation of the training and testing data, the data from the first 8 runs are used for training, while the last 2 runs are used for testing. Furthermore, we utilize 10% of the training data for validation purposes. To improve the model's generalization capabilities, we will shuffle the training data along with the corresponding training labels. The proposed network architecture is configured to run for 150 epochs, allowing sufficient learning from the training data. It employs a batch size of 32, which balances memory efficiency and stability in gradient updates. A learning rate of 0.001 is chosen to facilitate effective learning and prevent overshooting during optimization. Additionally, the weights and biases of the filters in each layer are trained jointly. To ensure robust evaluation, we determined trial-wise accuracy using a majority voting scheme. For each 5-s trial, we aggregated the predictions of its nine constituent windows, assigning the most frequent class label to the entire event.

We initially conduct binary classification to assess the model's discrimination ability corresponding to each movement pair, leading to 10 classifiers in total, and subsequently balance memory efficiency and stability in gradient updates. The five-class classification is implemented using a One vs. One (OvO) strategy. The final results of the five-class classification are obtained through a majority voting mechanism involving 10 binary classifiers. Each binary classifier cast a vote for its predicted class, and the class with the most votes was declared the winner. The class receiving the highest number of votes is selected as the final prediction. If two or more classes receive an equal number of votes, the tie is first addressed by conducting a sub-vote among the tied candidates. If the tie remains unresolved, the final decision is determined by aggregating the softmax class probabilities from the corresponding pairwise classifiers; specifically, the class with the highest total cumulative probability across all relevant binary comparisons is assigned as the final label. Each binary classification is trained for 150 epochs, resulting in a total of 1,500 epochs (150 × 10 classifiers).

### 2.5 Evaluation

We use the accuracy metric for classification results evaluation:


$$\begin{array}{l} \text{Accuracy (Acc)}=\frac{TP+TN}{TP+TN+FP+FN} \end{array}$$ (5)

where TP = True Positives, FP = False Positives, FN = False Negatives, and TN = True Negatives.

To provide a more detailed assessment of model performance, we also introduce the kappa statistic. Kappa contributes by quantifying the agreement between predicted and true classifications while accounting for chance agreement, thus offering insights into the model's reliability. Specifically, it highlights how well the model distinguishes between classes beyond random guessing, enabling a clearer evaluation of performance across both binary and multi-class scenarios.


$$\kappa =\frac{P_{o}-P_{e}}{1-P_{e}}$$

where the *P*_*o*_ is the observed agreement, representing the proportion of instances where the raters agree. *P*_*e*_ is the expected possibility, indicating the proportion of instances where agreement will be expected by chance.

To evaluate classification significance against the empirical chance level, a non-parametric permutation test was performed with 5,000 iterations, where class labels were randomly shuffled at the trial level. The empirical *p*-value was calculated as the proportion of permutations where the shuffled accuracy was greater than or equal to the observed accuracy. To evaluate the performance difference between models, we conducted a paired t-test on the results obtained from the binary classification of 10 finger pairs at three significance levels (*p* < 0.05, *p* < 0.01, and *p* < 0.001). Furthermore, the stability of the results was quantified by calculating 95% confidence intervals via a non-parametric bootstrap procedure across subjects. For each model, subject-wise accuracies were resampled with replacement 1,000 times to estimate the distribution of the mean accuracy.

## 3 Results and analysis

In this section, we present a comprehensive analysis of our findings, structured into four key subsections. First, we detail the classification performance of our proposed model. Second, we discuss the results of our network design. Third, we show the classification results of ablation studies. Finally, we report on experiments conducted with several state-of-the-art models applied to our dataset. Each subsection provides in-depth insights into our methodology and results, reinforcing the effectiveness of our proposed approach.

### 3.1 The results of the SCBAM

The binary and five-class classification results of individual fingers by the proposed SCBAM are presented in Table 1. The five subjects (S1 to S5) achieve accuracies of 77.38%, 79.40%, 79.69%, 78.57%, and 78.12% in binary classification, respectively, resulting in a mean accuracy of 78.63% (SD 1.56%; 95% CI [77.26, 80.00]%). Based on the results of binary classification, we observe that ten pairs of activities can be roughly categorized into three types: easy, moderate, and difficult.

**Table 1:** The classification results of the proposed network in accuracy.

**Binary classification**	**S1(%)**	**S2(%)**	**S3(%)**	**S4(%)**	**S5(%)**	**mean (SD) (%)**	***P***
Thumb vs. Index	83.99	83.38	85.08	83.33	82.71	83.70 (0.90)	**<0.001**
Thumb vs. Middle	83.66	85.17	83.38	81.67	83.05	83.39 (1.26)	**<0.001**
Thumb vs. Ring	84.74	85.76	83.39	84.66	86.44	85.00 (1.16)	**<0.001**
Thumb vs. Little	81.35	86.10	84.13	82.06	81.03	82.93 (2.14)	0.0041
Index vs. Middle	85.33	81.69	83.33	86.33	83.00	83.94 (1.87)	**<0.001**
Index vs. Ring	81.35	84.66	84.67	81.67	82.67	83.00 (1.59)	0.0035
Index vs. Little	72.53	77.00	77.89	78.04	77.89	76.67 (2.35)	0.0192
Middle vs. Ring	73.62	74.23	72.11	73.09	72.63	73.14 (0.83)	0.0241
Middle vs. Little	63.09	69.99	68.42	68.76	64.74	67.00 (2.93)	0.0339
Ring vs. Little	64.12	66.00	64.58	66.06	67.03	65.96 (1.46)	0.0376
**Average accuracy**	77.38	79.40	79.69	78.57	78.12	78.63 (1.56)	0.0122
**Five-class classification**	**S1(%)**	**S2(%)**	**S3(%)**	**S4(%)**	**S5(%)**	**mean (SD) (%)**	*P*
Accuracy	60.73	62.36	60.00	61.81	60.72	61.12 (0.95)	0.0423

The bold values represent the best classification performance for each respective action.

The six pairs from Thumb vs. Index to Index vs. Ring in Table 1 are categorized as easy type, with accuracies above 80%. The Index vs. Little and Middle vs. Ring are recognized as moderate type, with accuracies between 70% and 80%. Meanwhile, the Middle vs. Little and Ring vs. Little are considered as difficult types, with accuracies ranging from 60% to 70%. The classification results indicate varying levels of performance among the subjects, with S2 achieving the best performance. Moreover, all classification pairs for all subjects performed significantly above chance (*P* < 0.05). The thumb vs. the Ring finger performs the best overall at 85.00 (1.16%) in mean accuracy across five subjects. The finger pairs Thumb vs. Index, Thumb vs. Middle, Thumb vs. Ring, and Index vs. Middle achieve a highly significant result at the level of *P* < 0.001. In binary classification, finger pairs yield distinct results based on the included fingers: pairs including the Thumb (Thumb vs. Index, Thumb vs. Middle, Thumb vs. Ring, Thumb vs. Little) achieve an average accuracy of 83.76% across five subjects. Additionally, pairs including the index, middle, ring, and little fingers achieve average accuracies of 81.83%, 76.87%, 76.78%, and 73.14%, respectively, across the same subjects.

**Figure 5:**
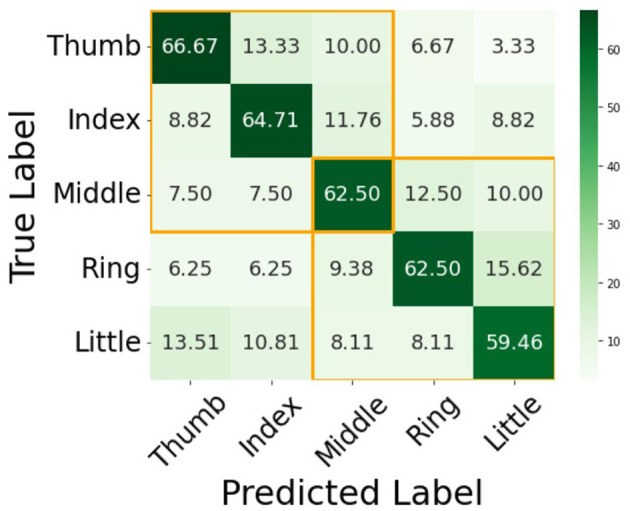
The confusion matrix of the five-class classification (%). The yellow rectangle in the top-left corner highlights the confusion between adjacent fingers (the Thumb, Index, and Middle fingers), indicating that these gestures are often misclassified. The yellow rectangle in the bottom-right corner highlights that the gestures of the adjacent fingers (Middle, Ring, and Little fingers), indicating that these gestures are often misclassified.

The five subjects (S1 to S5) achieve accuracies of 60.73%, 62.36%, 60.00%, 61.81%, and 60.72% in five-class classification, respectively, resulting in a mean accuracy of 61.12% (SD 0.95%; 95% CI [60.29, 61.95]%). From the confusion matrix depicted in Figure 5, the SCBAM achieves average accuracies of 66.67% for Thumb, 64.71% for Index, 62.50% for Middle, 62.50% for Ring, and 59.46% for Little fingers across five subjects. From this confusion matrix, it is clear that adjacent fingers tend to confuse with each other. For example, the thumb is misclassified as the index at a rate of 13.33%, and the ring finger is classified as the little finger at a rate of 15.62%. The confusion matrix reveals distinct patterns, indicating that the thumb, index, and middle fingers form one group with significant confusion tendencies, while the middle, ring, and little fingers constitute another one. The confusion clusters observed in Figure 5 (orange boxes) align with the somatotopic overlap in the primary motor cortex. Specifically, the confusion between the middle, ring, and little fingers reflects the known muscular and neural coupling termed enslavement, where these fingers share common multi-tendon muscle groups. This makes the neural signatures for these fingers more similar than those of the thumb and index finger. The higher classification accuracy for the Thumb (66.67%) and Index finger (64.71%) compared to the Little finger (59.46%) is consistent with the principle of cortical magnification. The thumb and index finger have larger, more specialized representations in the motor homunculus. Our SCBAM model effectively captures this increased neural real estate through its attention mechanism, resulting in more robust feature extraction for these digits.

**Figure 6:**
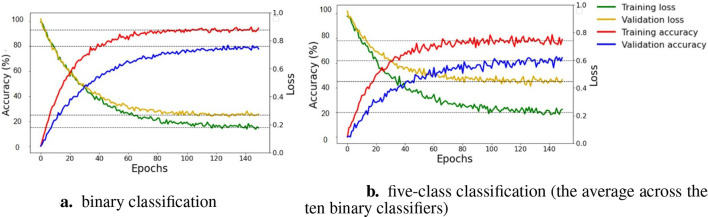
Training and validation learning curves for binary and five-class classification across epochs. The learning curve was measured using two metrics: accuracy on the left y-axis and loss on the right y-axis. In the binary classification scenario **(a)**, the graph displays training loss (yellow), validation loss (blue), training accuracy (green), and validation accuracy (red), with horizontal dashed lines marking accuracy thresholds (80% and 93%) and loss thresholds (0.15 and 0.25). The five-class classification scenario **(b)** presents analogous metrics, illustrating training loss (yellow), validation loss (blue), training accuracy (green), and validation accuracy (red), accompanied by accuracy thresholds (60% and 80%) and loss thresholds (0.20 and 0.40) to evaluate model performance.

The Figure 6 depicts the loss and accuracy curves for binary and five-class classification tasks on training and validation sets. For binary classification, Figure 6a shows that the training loss and validation loss decrease and converge to 0.15 and 0.25, respectively. The training accuracy rapidly increases during the first 40 epochs, ultimately converging at 93%, indicating robust learning from the training dataset. In parallel, the validation accuracy rises quickly over the first 45 epochs, stabilizing at 80%, which suggests a commendable ability to generalize, albeit with a notable gap compared to training performance. For five-class classification, Figure 6b presents the training loss and validation loss exhibit a downward trend, but stabilize at around 0.20 for training loss and approximately 0.45 for validation loss. The training accuracy reaches about 78%, while validation accuracy is approximately 62%. Overall, the figure supports the assertion that 150 epochs are ample for ensuring sufficient learning of the model, as indicated by the convergence of both accuracy and loss metrics. Continuing training beyond this point is unlikely to enhance performance and may lead to overfitting.

As our study includes both binary classification and five-class classification, we employ the Kappa statistic as a complementary measure to accuracy, providing a more nuanced classification result by accounting for chance agreement. The results are shown in Table 2. It achieves an average Kappa of 0.7898 for binary classification across 10 pairs in five subjects. For five-class classification, the average Kappa is 0.6130.

**Table 2:** The classification results of the proposed model in Kappa.

**Binary classification**	**S1**	**S2**	**S3**	**S4**	**S5**	**Mean**
Thumb vs. index	0.8312	0.8298	0.8501	0.8250	0.8225	0.8217
Thumb vs. middle	0.8350	0.8199	0.8317	0.8123	0.8205	0.8239
Thumb vs. ring	0.8403	0.8612	0.8120	0.8340	0.8605	0.8416
Thumb vs. little	0.8107	0.8172	0.8285	0.8265	0.8080	0.8182
Index vs. middle	0.8476	0.8071	0.8275	0.8369	0.8064	0.8251
Index vs. ring	0.7964	0.8372	0.8174	0.8035	0.8133	0.8136
Index vs. little	0.7280	0.7535	0.7565	0.7400	0.7681	0.7512
Middle vs. ring	0.7196	0.7248	0.7035	0.7167	0.7162	0.7162
Middle vs. little	0.6390	0.6643	0.6725	0.6673	0.6280	0.6542
Ring vs. little	0.6375	0.6219	0.6405	0.6408	0.6790	0.6434
**Average kappa**	0.7785	0.7937	0.7940	0.7903	0.7923	0.7898
**Five-class classification**	**S1**	**S2**	**S3**	**S4**	**S5**	**Mean**
Accuracy	0.6174	0.6306	0.5987	0.6053	0.6129	0.6130

### 3.2 The results of network design

**Figure 7:**
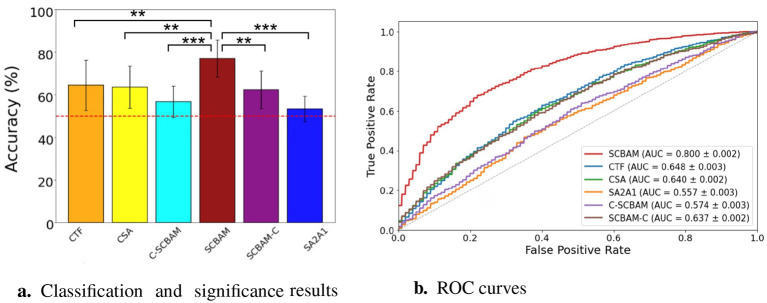
The classification and significance test results and ROC curves of SCBAM with the model variants in network design. **(a)** The classification and significantce test results of the SCBAM and its network design models. **(b)** The ROC curves of the network design. ^***^represents the *P* < 0.001, ^**^denotes 0.001 < *P* < 0.01, ^*^ is 0.01 < *P* < 0.05. The red dashed line is the chance level.

In our network design, we conduct a thorough study of the parameter settings, including the number of convolutional layers, the number of attention modules, and the order of attention module placement. By comparing the average accuracy across five subjects from different model candidates reported in Section 2, we identify the CBAM as the most suitable attention mechanism for our classification task. First, the self-attention mechanism between the initial and the final convolutional layer (CSA) model achieves an average accuracy of 63.98% for binary classification and 58.95% for five-class classification. Next, two convolutional layers integrating with a CBAM module in sandwich shape (SCBAM) model achieve an accuracy of 78.63% for binary classification and 61.12% for five-class classification. Integrating with a multi-head transformer (CTF) achieves an average accuracy of 64.89% for binary classification and 59.34% for five-class classification. Results from these three models indicate that embedding the CBAM module yields the best classification accuracy for our task. Switching the two attention mechanisms results in a decrease of 23.00% in binary classification accuracy and a drop of 24.40% in five-class classification accuracy. These results reveal that the order of attention modules is significant in network design. Attempts to add extra convolutional layers before and after the SCBAM model (C-SCBAM and SCBAM-C) all resulted in a drop in classification accuracy as reported in Table 3. These findings demonstrate that having one layer before the attention modules and one layer after is sufficient for our task. Excessive layers led to a model that was too complex for the limited training data, resulting in overfitting. Besides, the significance test of the binary classification results for all variants in the network design is presented in Figure 7a, the ROC curves further illustrate the trade-offs between sensitivity and specificity for each model as depicted in Figure 7b.

**Table 3:** The average accuracy of model candidates across five subjects.

**Binary classification**	**SCBAM (%)**	**CTF (%)**	**CSA (%)**	**SA2A1 (%)**	**C-SCBAM (%)**	**SCBAM-C (%)**
Thumb vs. index	**83.70**	81.01	74.24	56.95	69.31	71.58
Thumb vs. middle	**83.39**	61.03	59.31	48.97	69.27	72.06
Thumb vs. ring	**85.00**	67.12	55.93	56.61	54.13	70.53
Thumb vs. little	82.93	**86.44**	82.37	54.58	52.54	69.47
Index vs. middle	**83.94**	48.47	54.24	55.25	63.05	67.89
Index vs. ring	**83.00**	73.33	60.00	54.00	52.20	65.15
Index vs. little	**76.67**	63.00	70.00	54.00	57.28	56.91
Middle vs. ring	**73.14**	58.31	72.88	56.95	54.67	58.45
Middle vs. little	**67.00**	50.50	50.85	56.61	49.83	53.09
Ring vs. little	**65.96**	59.66	60.00	56.33	52.66	51.08
**Average accuracy**	**78.63**	64.89	63.98	55.63	57.49	63.62
**Five-class classification**	**61.12**	59.34	58.95	36.72	48.58	48.95

CTF, the transformer module between the first and the second convolutional layers; CSA, the self-attention mechanism between the first and the second convolutional layers; SA2A1, the first convolutional layer + spatial and rhythm attention + filters attention + the second convolutional layer; C-SCBAM, add an extra convolutional layer before the SCBAM; SCBAM-C, add an extra convolutional layer after the SCBAM. The bold values represent the best classification performance for each respective action.

### 3.3 The ablation studies of the proposed network

The ablation studies of SCBAM performed across five subjects are reported in Table 4, involving a series of model variants. By removing two convolution layers, only the CBAM module achieves an average decrease in accuracy of 16.94% for the binary classification task and a decrease of 12.55% for the five-class classification task compared to the SCBAM. This significant decrease in accuracy (at a level of <0.001) illustrates that the two convolutional layers also play a crucial role in performance, although not as profound as that of the CBAM module. By removing the first convolutional layer, the CBAM model shows an average accuracy decrease of 15.12% for the binary classification task compared to the proposed model. Furthermore, for the five-class classification, removing the first convolutional layer results in an accuracy drop of 4.93%. The last convolutional layer also plays an important role, as its removal leads to a drop of 15.90% for the binary classification and a decrease of 1.12% for the five-class classification task. By conducting a t-test between the two model variants and the proposed SCBAM, the results indicate that both individual convolutional layers are significant contributors to the model's performance. By removing the attention modules, the two convolution layers (two convs) yield a value of 28.10% for five-class classification, whereas binary classification values range from 46.32% to 65.76%. This results in an average accuracy decrease of 21.62% for binary classification and 33.02% for five-class classification compared to the proposed model. These results demonstrate that the CBAM plays a crucial role in enhancing the model's performance. By removing the second attention module, the SA1 shows an average accuracy decline of 16.87% for the binary classification and a decrease of 9.57% for the five-class classification compared to SCBAM. By removing the first attention module, the SA2 model exhibits an average accuracy decrease of 9.77% for the binary classification and a decrease of 2.67% for the five-class classification compared to the proposed network. From the accuracy comparison, the first attention mechanism has the weakest effect on classification performance. This may be due to the fact that it operates in the filter domain generated by the preceding convolutional layer, meaning it refines the obtained features rather than extracting new features from the data. On the contrary, the second attention mechanism has a greater effect on the model than the other modules. This is because it is the key component for handling rhythm domain information, which is not addressed by any other modules.

**Table 4:** The results of ablation studies across five subjects.

**Binary classification**	**SCBAM**	**fCBAM**	**CBAMs**	**SA1**	**SA2**	**CBAM**	**two convs**	**NFBCSP**	**NCAR**
Thumb vs. index	**83.70**	78.01	74.67	66.90	75.90	73.46	61.34	64.12	69.45
Thumb vs. middle	**83.39**	72.12	69.18	62.14	71.58	73.75	65.76	56.91	69.39
Thumb vs. ring	**85.00**	67.38	59.96	67.59	77.95	75.36	62.63	62.06	63.62
Thumb vs. little	**82.93**	66.34	72.03	65.52	72.82	63.36	59.47	55.15	64.74
Index vs. middle	**83.94**	67.84	64.31	66.00	75.38	63.57	56.84	53.09	68.42
Index vs. ring	**83.00**	62.83	70.00	64.83	74.00	64.59	59.47	52.06	61.72
Index vs. little	**76.67**	60.00	58.73	59.47	69.33	54.00	58.95	51.72	60.03
Middle vs. ring	**73.14**	48.13	62.33	56.19	65.33	44.59	48.89	53.09	52.56
Middle vs. little	**67.00**	50.00	53.40	56.91	54.74	52.39	46.32	51.03	51.43
Ring vs. little	**65.96**	54.64	50.52	52.06	51.55	51.83	50.50	48.28	51.72
**Average accuracy**	**78.63**	62.73	63.51	61.76	68.86	61.69	57.01	54.75	61.31
**Five-class classification**	**61.12**	60.00	56.19	51.55	58.45	48.57	28.10	49.09	53.18

fCBAM, the first convolutional layer with the CBAM module; CBAMs, the CBAM module alongside the second convolutional layer; SA1, two convolution layers (sandwich) and filters attention; SA2, two convolution layers (sandwich) and spatial and rhythm attention; CBAM, only Convolutional Block Attention Module; two convs, only two convolution layers; NFBCSP, the proposed method without FBCSP; NCAR, the proposed method without CAR. The bold values represent the best classification performance for each respective action.

**Figure 8:**
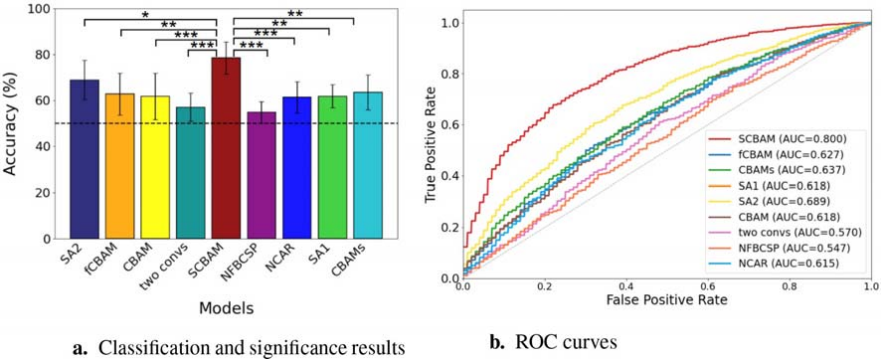
The classification and significance test results and ROC curves of SCBAM with the ablation variants in the pipeline. **(a)** The classification and significance test results of the SCBAM and ablation variants. **(b)** The ROC curves of the SCBAM and ablation variants. *** represents the *P* < 0.001, ** denotes 0.001 < *P* < 0.01, * is 0.01 < *P* < 0.05. The red dashed line is the chance level.

Besides, ablation experiments are conducted to verify the effectiveness of FBCSP and CAR. Compared to the proposed method, if we remove the FBCSP from the data processing pipeline, the accuracy drops by 23.88% in binary classification and 12.03% in five-class classification. If we remove the CAR, the accuracy declines by 17.32% in binary classification and by 7.94% in five-class classification, respectively. These results confirm that both steps are indispensable for achieving optimal classification performance. It is worth noting that, through the significance test of the binary classification results for all variants within the proposed SCBAM data pipeline, as presented in Figure 8a, none of the modules or processing steps can be removed, as they all play significant roles in the final performance at a level of <0.01. Besides, the ROC curves further validate the trade-offs between sensitivity and specificity for each model as illustrated in Figure 8b.

### 3.4 The results of comparasion experiments

**Figure 9:**
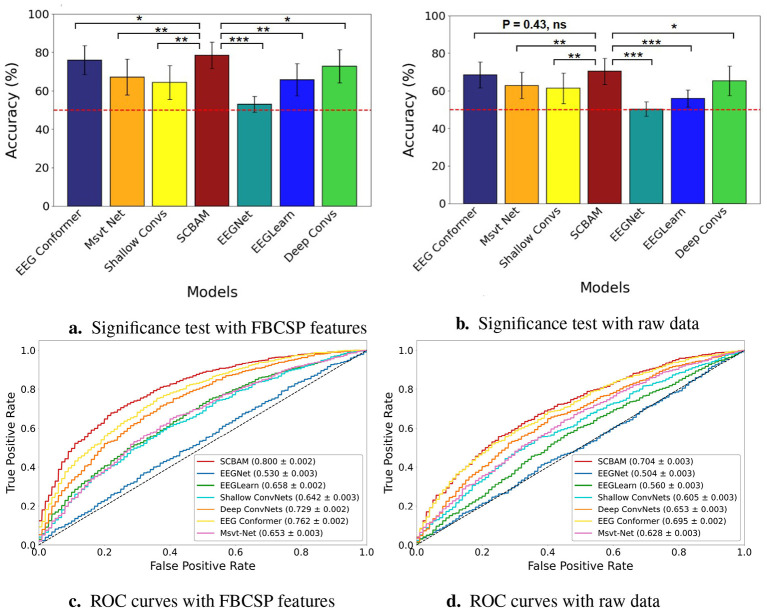
The classification and significance test results and ROC curves of the SCBAM and the other six benchmark models with FBCSP features and raw data. **(a)** The classification results and significance test of the SCBAM and the other six models with FBCSP features. **(b)** The classification results and significance test of the SCBAM and the other six models with raw data. **(c)** The ROC curves of the SCBAM and the other six models with FBCSP features. **(d)** The ROC curves of the SCBAM and the other six models with raw data. *** represents the *P* < 0.001, ** denotes 0.001 < *P* < 0.01, * is 0.01 < *P* < 0.05. The red dashed line is the chance level.

**Figure 10:**
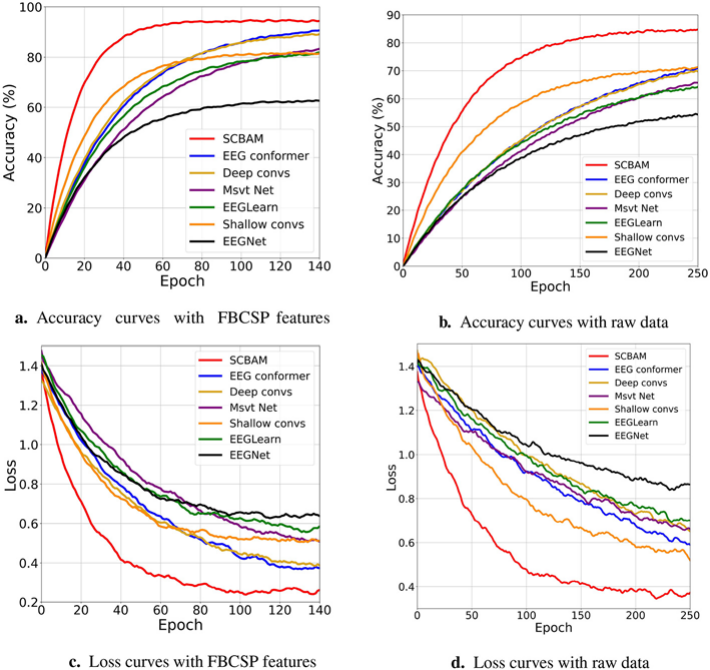
The learning curves of the SCBAM and the other six benchmark models with FBCSP features and raw data. **(a)** Accuracy curves with FBCSP features. **(b)** Accuracy curves with raw data. **(c)** Loss curves with FBCSP features. **(d)** Loss curves with raw data.

We compare the classification performance of the proposed network with state-of-the-art models, including EEGNet, EEGLearn, Shallow ConvNets, Deep ConvNets, EEG Conformer, and Msvt Net with FBCSP features and raw data independently. The classification results with FBCSP features are presented in Table 5. For binary classification, SCBAM exceeds the performance of the EEGNet, EEG Learn, Shallow ConvNets, Deep ConvNets, EEG Conformer, and Msvt Net by 25.69%, 12.93%, 14.37%, 13.31%, 2.59%, and 13.25%, respectively. A paired *t*-test is conducted to assess the significance of the differences in binary classification accuracy between the proposed model and six benchmark models, as demonstrated in Figure 9a. Specipically, the SCBAM surpasses the EEG Conformer and Deep ConvNets at the 0.05 significance level. The SCBAM significantly outperforms EEGLearn, Shallow ConvNets, and Msvt Net at the 0.01 level. SCBAM exhibits a highly significant performance advantage over EEGNet at the 0.001 level. For five-class classification, SCBAM achieves an average classification accuracy of 61.12%, while the EEGNet, EEGLearn, Shallow ConvNets, Deep ConvNets, EEG Conformer, and Msvt Net exhibit accuracies in the range from 45% to 58%. The ROC curves further illustrate the trade-offs between sensitivity and specificity for each model as presented in Figure 9c. The accuracy and loss learning curves during training are depicted in Figures 10a, c. The accuracy of SCBAM rises sharply until it reaches 94.00% around the 45th epoch, surpassing benchmark models in both convergence speed and final accuracy. Subsequently, the accuracy of EEG Conformer and Deep ConvNets stabilizes around 90% at 120th epoch. While Shallow ConvNets, EEG Learn, and Msvt Net acheive approximately 80% accuracy at the 120th epoch. Among the six benchmark models, Shallow ConvNets converge most rapidly, reaching stability by the 80th epoch. The loss of SCBAM exhibits a sharp decline during the first 40 epochs, reaching a low value of approximately 0.25 by the 80th epoch. The loss of EEG Conformer and Deep ConvNets reaches a turning point around the 60th epoch, achieving a low value of approximately 0.4 by the 120th epoch. Shallow ConvNets converges more rapidly reaching its stability loss value of approximately 0.5 by the 100th epoch. The results indicate that SCBAM significantly surpasses the performance and efficiency of these six models.

**Table 5:** Classification results of proposed model against state-of-the-art techniques with FBCSP features.

**Binary classification**	**SCBAM**	**EEGNet (Lawhern et al., 2018)**	**EEG learn (Bashivan et al., 2016)**	**Shallow convs (Schirrmeister et al., 2017)**	**Deep convs (Schirrmeister et al., 2017)**	**EEG conformer (Song et al., 2023)**	**Msvt net (Liu et al., 2024)**
Thumb vs. index	**83.70**	50.52	72.58	72.11	81.90	82.76	79.44
Thumb vs. middle	**83.39**	54.14	73.68	71.58	82.76	81.90	77.54
Thumb vs. ring	**85.00**	52.54	71.58	73.62	78.42	83.05	76.58
Thumb vs. little	**82.93**	51.19	70.53	72.60	79.90	82.11	73.09
Index vs. middle	**83.94**	62.03	69.47	68.95	76.58	81.69	72.00
Index vs. ring	**83.00**	48.67	72.06	67.37	73.62	77.89	64.12
Index vs. little	**76.67**	58.33	64.12	58.95	70.53	73.68	63.12
Middle vs. ring	**73.14**	51.86	63.09	59.47	69.47	71.58	62.58
Middle vs. little	**67.00**	47.80	49.48	50.00	58.11	63.12	52.14
Ring vs. little	**65.96**	52.33	51.38	48.95	57.38	62.58	53.21
**Average accuracy**	**78.63**	52.94	65.70	64.26	72.87	76.04	65.38
**Five-class classification**	**61.12**	45.68	47.52	53.26	56.73	58.55	54.00

The bold values represent the best classification performance for each respective action.

The classification results with raw data are summarized in Table 6, SCBAM consistently outperforms benchmark models across both binary and five-class classification tasks. In binary classification, SCBAM achieves accuracy improvements of 20.06%, 14.42%, 9.95%, 5.03%, 0.99%, and 7.47% over EEGNet, EEGLearn, Shallow ConvNets, Deep ConvNets, EEG Conformer, and Msvt Net, respectively. In the five-class task, SCBAM surpasses these benchmark models by 18.03%, 16.50%, 13.13%, 9.12%, 4.61%, and 10.99%, respectively. A paired *t*-test is conducted to assess the significance of the differences in binary classification accuracy between the proposed model and six benchmark models, the results are demonstrated in Figure 9b. SCBAM achieves highly significant improvements over EEGNet and EEGLearn (*P* < 0.001), and significantly outperforms Shallow ConvNets and Msvt Net (*P* < 0.01) and Deep ConvNets at the 0.05 significance level. It is worth noting that the EEG Conformer demonstrates strong classification performance that is statistically comparable to our proposed SCBAM (*P* = 0.43). The ROC curves further illustrate the trade-offs between sensitivity and specificity for each model as presented in Figure 9d. Besides, the accuracy and loss learning curves during training are depicted in Figures 10b, d. SCBAM stabilizes at an accuracy of 81% around 130th epoch and a steady loss value of 0.4 around 150th epoch, outperforming other models.

**Table 6:** Classification results of proposed model against state-of-the-art techniques with raw data.

**Binary classification**	**SCBAM**	**EEGNet (Lawhern et al., 2018)**	**EEG learn (Bashivan et al., 2016)**	**Shallow convs (Schirrmeister et al., 2017)**	**Deep convs (Schirrmeister et al., 2017)**	**EEG conformer (Song et al., 2023)**	**Msvt net (Liu et al., 2024)**
**Thumb vs. index**	**78.42**	**51.05**	**60.51**	**69.61**	**72.53**	**76.58**	**70.00**
Thumb vs. middle	73.09	48.89	54.13	70.32	71.03	**74.55**	68.58
Thumb vs. ring	**79.44**	52.50	61.18	70.78	70.52	74.84	69.47
Thumb vs. little	**74.12**	51.05	52.54	69.58	72.06	71.48	66.74
Index vs. middle	**72.26**	56.84	62.03	63.09	72.00	72.09	68.05
Index vs. ring	**70.54**	50.50	58.66	61.38	69.38	69.19	65.43
Index vs. little	**71.05**	50.00	58.33	52.50	61.03	66.74	61.05
Middle vs. ring	**68.75**	53.68	52.33	56.84	61.04	65.43	57.37
Middle vs. little	**59.38**	46.32	51.86	51.05	51.38	58.47	51.53
Ring vs. little	**56.42**	42.11	47.79	48.89	52.20	54.26	50.53
**Average accuracy**	**70.35**	50.29	55.93	60.40	65.32	69.36	62.88
**Five-class classification**	**56.75**	38.72	40.25	43.62	47.63	52.14	45.76

The bold values represent the best classification performance for each respective action.

In summary, the classification results of the SCBAM and benchmark models with FBCSP features significantly outperformed those obtained from raw data. This underscores the importance of integrating well-established features with deep learning networks, as it facilitates a balance between performance and efficiency. Consequently, we investigated the efficiency analysis of the seven models with FBCSP features. As described in Section 2.4, we conduct 2,250 trials from 10 experimental runs, where 1,800 trials from the first 8 runs are used for training, and 450 trials from the last 2 runs are used for testing. The assignment of training and test data is consistent for both binary and five-class classification scenarios. It is worth noting that the FBCSP step takes 314 s (around 5 min). The time consumption of seven models are indicated in Table 7. For binary classification, SCBAM exhibits the most efficient performance with a per-epoch time of 1 s, resulting in a total training time of 25 min and a testing time of just 10 s. Shallow ConvNets, though slightly slower with a per-epoch time of 1.8 s, maintain a competitive training time of 45 min and a testing time of 14 s. Deep ConvNets demonstrate a more pronounced increase in computational demands, featuring a per-epoch time of 3 s and a total training time of 74.5 min, with a testing time of 28 s. EEG Conformer performs a per-epoch time of 3.3 s and a total training time of 80 min, with a testing time of 24 s. EEGNet, Msvt Net, and EEGLearn exhibit even greater time consumption. For five-class classification, SCBAM exhibits the most efficient performance with a per epoch time of 1.5 s, resulting in a total training time of 37.5 min ((150 × 10 epochs × 1.5 s) / 60) and a testing time of just 17 s, which is lower compared to the other six models. This analysis underscores SCBAM's efficiency, making it the optimal choice for scenarios requiring quick training and inference times. It is worth noting that both the SCBAM and Shallow ConvNets have two convolutional layers. Deep ConvNets, EEG Conformer, EEGNet, EEGLearn, and Msvt Net rely on a larger number of convolutional layers and deeper networks for feature extraction, require significantly longer training times.

**Table 7:** The runtime of all models with FBCSP features.

**Binary classification**	**Per epoch time (s)**	**Training time (min)**	**Testing time (s)**
SCBAM	1	25	10
Shallow ConvNets	1.8	45	14
Deep ConvNets	3	74.5	28
EEG Conformer	3.2	80	24
EEGNet	3.5	87.5	25
Msvt Net	4	100	30
EEGLearn	4.2	105	34
**Five-class classification**	**Per epoch time (s)**	**Training time (min)**	**Testing time (s)**
SCBAM	1.5	37.5 (10 classifiers)	17
Shallow ConvNets	2.4	60 (10 classifiers)	19
Deep ConvNets	3.3	82.5 (10 classifiers)	33
EEG Conformer	4.3	107.5 (10 classifiers)	29
EEGNet	4.5	112.5 (10 classifiers)	42
Msvt Net	4.8	120 (10 classifiers)	50
EEGLearn	5	125 (10 classifiers)	56

Comparing the time consumed in the binary and five-class classification scenarios of binary and five-class classification, it is evident that the five-class classification always consumes longer time in each epoch and total training and test time. This is due to the one-vs-one strategy employed in the five-class classification. In this scenario, we utilize 10 binary classifiers for each trial, leading to a cumulative processing time that may suggest a 10-fold increase relative to a single binary classification. In contrast, the binary classification task only requires distinguishing between two classes, which necessitates computational resources for just one binary classifier. Additionally, in the binary classification framework, each movement is represented in four pairs. For instance, the thumb is compared against the index, middle, ring, and little fingers. Consequently, each trial is read and classified four times overall. In summary, in five-class classification, each data trial is read once but classified 10 times using binary classifiers. In contrast, in the binary classification scenario, the data for each trial is read four times and classified through four different binary classifiers. These factors contribute to the observation that while the training time for five-class classification is longer than that for binary classification, it is not as extensive as 10 times the duration of binary classification.

## 4 Discussion

In this section, we summarize the main contributions of this study and discuss our results in relation to those reported in the current literature. In addition to analyzing the individual finger classification outcomes using the uHD EEG dataset, we also consider results from EEG and ECoG data. By contextualizing our findings within the broader landscape of brain signals, we aim to highlight the advantages and limitations of uHD EEG, paving the way for future research in this area. At the same time, we examine the decoding algorithms utilized in this field over the past two decades and identify emerging trends, such as the integration of deep learning modules. Our study suggests a promising direction aimed at enhancing classification accuracy while managing computational complexity, ensuring that computation times remain sufficiently short for online experiments and real-time applications.

### 4.1 Individual finger classification with electrical brain signals

**Figure 11:**
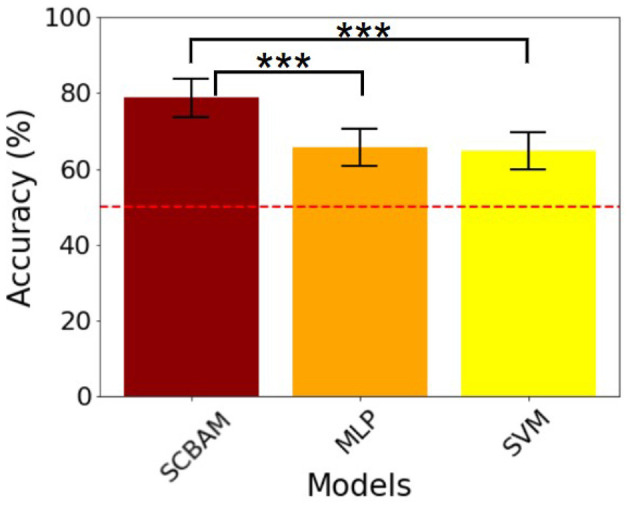
The classification and significance test results of the SCBAM and literature models on uHD EEG. 1. ^***^ represents the *P* < 0.001, ^**^ denotes 0.001 < *P* < 0.01, ^*^ is 0.01 < *P* < 0.05. 2. The red dashed line is the chance level.

First, we compare our results with those from recent literature that utilized the same uHD EEG dataset, including one study that employed an SVM classifier (Lee et al., 2022) and another that used an MLP (Nemes et al., 2024). The results are summarized in the Table 8, indicating varying levels of accuracy across different finger pairs. The SCBAM method consistently outperforms these two approaches, achieving the highest average accuracy of 78.63% across all finger pairs and subjects. In contrast, the SVM and MLP methods recorded average accuracies of 64.8% and 65.68%, respectively. As depicted in Figure 11, statistical analysis using the t-test reveals that SCBAM significantly improved performance compared to both SVM and MLP at a significance level of 0.001. These findings highlight the accuracy of the SCBAM achieved for individual finger binary classification, suggesting it as a superior choice for this task. One thing to note is that in both studies, the ring finger is reported to have greater accuracy and has been regarded as easier to distinguish from the others. This is attributed to the fact that the four movement pairs, including the ring finger, rank among the top four in accuracy of all ten finger pairs in their results, which was also inferred in Table 1 in (Lee et al. 2022) and Table 3 in (Nemes et al. 2024). However, our results indicate that the ring finger does not stand out significantly. Instead, it is more reasonable to assert that the thumb and index fingers demonstrate greater classification accuracy in our findings. The binary classification between five individual fingers with EEG data has also been reported in (Liao et al. 2014). Their findings indicate an average accuracy of 77.11%. In their results, even though the Ring vs. Little pair achieves the highest accuracy, the Ring vs. Thumb, Ring vs. Index, and Ring vs. Middle pairs are ranked 5th, 7th, and 9th in all ten pairs, respectively [as indicated in Table 2 in Liao et al. (2014)]. The overall classification performance of the ring finger pairs appears to be less pronounced.

**Table 8:** uHD EEG classification results from current literature and proposed model in accuracy (mean (SD) %).

**Data**	uHD EEG			**EEG**
**Activity**	**SCBAM**	**MLP (Nemes et al., 2024)**	**SVM (Lee et al., 2022)**	**SVM (Liao et al., 2014)**
Thumb vs. Index	**83.70 (0.90)**	64.00 (6.50)	63.30 (3.50)	71.30 (13.30)
Thumb vs. Middle	**83.39 (1.26)**	65.00 (5.50)	64.00 (4.10)	74.30 (1.14)
Thumb vs. Ring	**85.00 (1.16)**	72.40 (10.10)	69.80 (7.10)	77.60 (12.60)
Thumb vs. Little	**82.93 (2.14)**	62.40 (2.90)	61.80 (3.40)	78.20 (13.50)
Index vs. Middle	**83.94 (1.87)**	62.80 (4.10)	60.20 (4.40)	69.20 (13.60)
Index vs. Ring	**83.00 (1.59)**	65.60 (13.40)	68.60 (9.70)	73.50 (8.80)
Index vs. Little	**76.67** (2.35)	60.60 **(1.50)**	61.80 (5.00)	79.60 (13.30)
Middle vs. Ring	**73.14 (0.83)**	69.60 (7.50)	70.60 (9.40)	71.20 (12.20)
Middle vs. Little	**67.00** (2.93)	62.80 **(2.40)**	62.60 (2.60)	77.90 (14.10)
Ring vs. Little	65.96 **(1.40)**	**70.40** (9.30)	65.20 (3.90)	80.20 (10.80)
**Average accuracy**	**78.63 (1.56)**	65.52 (6.32)	64.80 (5.31)	75.30 (11.33)

The bold values represent the best classification performance for each respective action.

A few studies have reported the five-class classification of individual fingers, particularly using ECoG signals. From the study of Kubanek (Kubánek et al., 2009), the five-class individual finger classification achieved 90.6%, 89.9%, 76.4%, 68.1%, and 76.7% for their five subjects, respectively, with an average of 80.3 (8.66)% across subjects. Chestek et al. (2013) employed a Naive Bayes classifier, which achieved classification accuracies of 74%, 98%, and 66% for each subject, with an average of 79.33 (13.49)% across three subjects. Their study emphasizes the effect of gamma band features. Both of these studies utilize macro ECoG signals, which have a spatial resolution of around 10 mm. For Hotson et al. (2016), who employed a micro ECoG with an electrode resolution of 2.3 mm, they achieved a five-class classification accuracy of 76% using Linear Discriminant Analysis (LDA) with only one subject. However, there are currently no literature reports on five-class classification results of individual finger flexion and extension tasks using uHD EEG data. In our study, we achieved five-class classification accuracies of 60.73%, 62.36%, 60.00%, 61.81%, and 60.72% for our five subjects, respectively. This results in an average accuracy of 61.12% with a small standard deviation of 0.95% across subjects. Compared to ECoG, our classification accuracy is lower; however, it is important to highlight that our results exhibit greater stability across subjects. The high variance observed in ECoG decoding is likely due to the cantotomy surgery, which is primarily designed to address clinical situations. This often results in electrode placements varying significantly among individuals, leading to configurations that may be either far from or close to the motor cortex. In contrast, uHD EEG collection does not depend on clinical surgery for electrode implantation, allowing for convenient electrode placements and calibrations. This property contributes to the stable results observed in our study. Xiao and Ding (2013) also demonstrated that with EEG signals, five-class classification reached an accuracy of 45.2% using three principal component features. The significant difference in accuracy between uHD EEG and EEG data underscores that spatial resolution is a crucial factor for individual finger decoding. What's more, from our confusion matrix and the confusion matrix derived from ECoG results, it is evident that adjacent fingers tend to confuse each other, while fingers that are farther apart are easier to distinguish. This aligns with the common understanding: fingers that are distanced from each other have less overlapping musculature and are less likely to interfere with one another during movement. Additionally, their corresponding cortical areas are situated farther apart, making them comparatively easier to differentiate in classification tasks. The observed grouping of the Thumb, Index, and Middle fingers in Figure 5 reflects both the common innervation by the median nerve and the functional synergies required for precision handling (Schieber and Santello, 2004). Conversely, the confusion between the Ring and Little fingers aligns with ulnar nerve distribution and the enslavement phenomenon, where peripheral mechanical and neural coupling limit independent digit control (Häger-Ross and Schieber, 2000). Our model's higher accuracy for the thumb and index is further supported by the principle of cortical magnification, as these digits possess significantly larger and more complex representations in the motor cortex (Schieber, 2001). This finding is crucial for understanding the underlying neural mechanisms of finger movements.

### 4.2 Deep neural network application

In our study, we design a Sandwich-enhanced CBAM (SCBAM) network that integrates convolutional layers with a CBAM module. The two attention mechanisms in CBAM process information from different domains for uHD EEG signals. The filter attention highlights time-frequency patterns, prioritizing the most informative frequency bands relevant to finger movements. Spatial attention focuses on the most active electrodes, enhancing sensitivity to subtle differences in motor patterns. The multidimensional feature extraction capability of the attentional mechanisms in CBAM aligns well with the multi-domain features of uHD EEG signals, leading to significant improvements in both performance and efficiency. This is also validated by the results in Section 3.4, the proposed SCBAM model shows significant improvements in performance over several state-of-the-art models, including EEGNet, EEGLearn, Shallow ConvNets, Deep ConvNets, EEG Conformer, and Msvt Net in both binary and five-class classification tasks, Tables 5, 6. It also requires much less inference time compared to these models in Table 7. This is due to the attention mechanisms in SCBAM, which are also validated in ablation studies.

The classification results indicate that while convolutional layers are highly effective at extracting multi-level features for movement discrimination, they eventually reach a performance saturation point. Simply increasing the depth of the model, as seen in architectures like EEGNet (Lawhern et al., 2018) or EEGlearn (Bashivan et al., 2016), does not necessarily lead to breakthroughs, creating a performance upper bound for both binary and five-class classification. The introduction of attention mechanisms, such as those in the EEG Conformer (Song et al., 2023), represents a strategic shift in model design. These mechanisms operate along the preceding convolutional filter domain to enhance both classification performance and computational efficiency. It is worth noting that the EEG Conformer demonstrates a strong classification ability that is statistically comparable to the proposed SCBAM (*P* = 0.43) when fed with raw data. However, the proposed SCBAM further refines this approach by embedding dual attention modules to manage innovative discriminative features from the filters, spatial, and rhythm domains. This dual attention module plays a crucial role in automatically extracting discriminative features from the input data in the proposed SCBAM network. The input of the proposed SCBAM network is the features extracted by the Filter Bank Common Spatial Pattern (FBCSP). These features have been thoroughly studied for decades (Lotte and Guan, 2011), and it is widely accepted that they are key features in neural decoding. We drew the inspiration of combining traditional features with neural networks from the Shallow ConvNets proposed by Tonnio Ball (Schirrmeister et al., 2017). This two-step design integrates established signal processing principles with the data-driven capabilities of deep neural networks, resulting in a lightweight architecture that achieves statistically significant results in individual finger classification. While the five-class accuracy of 61.3% notably above the 20% chance level, highlights the inherent difficulty of decoding fine-grained finger movements, our analysis of the error structure reveals that misclassifications are primarily concentrated among anatomically adjacent fingers. This performance provides a robust foundation for future dexterous BCI research, where these specific decoding patterns can be further refined through larger cohorts and online experiment.

### 4.3 Future study

While the current study utilizes an open-access dataset (Lee et al., 2022) consisting of five subjects. Our laboratory is currently initiating experiments using identical equipment and experimental paradigms with more than 20 subjects. This expansion will facilitate a more comprehensive investigation of inter-subject variability. Furthermore, we plan to bridge the gap between offline classification and practical utility by transitioning to closed-loop online experiments. Future research will also prioritize optimizing real-time performance metrics, such as Information Transfer Rate (ITR) and system latency, to meet the benchmarks necessary for intuitive and responsive BCI control.

## 5 Conclusion

In this study, we propose a Sandwich enhanced CBAM (SCBAM) network for decoding individual finger movements utilizing the uHD EEG dataset collected by g. Pangolin system, achieving an average accuracy of 78.63 (1.56)% in binary classification and 61.12 (0.95)% in five-class classification. The results significantly outperform the existing methods of SVM and MLP for binary finger classification with the same dataset. Comparative experiments are conducted between our proposed model and several state-of-the-art neural networks, including EEGNet, EEGLearn, Shallow ConvNets, and Deep ConvNet. These results indicate that the hybrid model incorporating attention mechanisms with convolutional layers is an effective way to improve performance for the task of individual finger classification.

From the network design process and the ablation experiments, the proposed SCBAM model can break the accuracy saturation of networks that consist only of convolutional layers. Combined with a well-established FBCSP in the BCI field, SCBAM takes full advantage of the brain rhythms and spatial patterns. This combination allows the network to maintain a small number of parameters while ensuring satisfactory classification performance.

Compared to the EEG signals for the individual finger task, uHD EEG shows significant improvements in both binary and five-class classification, attributed to its high spatial resolution. In comparison with ECoG signals, uHD EEG exhibits comparatively lower accuracy for five-class classification. However, uHD EEG demonstrates surprising stability, with the five subjects achieving a small standard deviation of 0.95%. This stability is attributed to the flexibility of electrode placement and the convenience of calibration during experiments, both of which are crucial for real-life scenarios. In conclusion, this study proposes a lightweight yet highly effective network for individual finger classification, emphasizing the potential of uHD EEG for real-time dexterous tasks in the field of brain-computer interfaces (BCIs).

## Data Availability

Publicly available datasets were analyzed in this study. This data can be found here: https://osf.io/4dwjt?view_only=d23acfd50655427fbaae381a17cbfbcc. Repository: OSF (Open Science Framework).
